# Smart waste classification in IoT-enabled smart cities using VGG16 and Cat Swarm Optimized random forest

**DOI:** 10.1371/journal.pone.0316930

**Published:** 2025-02-28

**Authors:** Akshat Gaurav, Brij Bhooshan Gupta, Varsha Arya, Razaz Waheeb Attar, Shavi Bansal, Ahmed Alhomoud, Kwok Tai Chui

**Affiliations:** 1 Ronin Institute, Montclair, New Jersey, United States of America; 2 Department of Computer Science and Information Engineering, Asia University, Taichung, Taiwan; 3 Department of Medical Research, China Medical University Hospital, China Medical University, Taichung, Taiwan; 4 Symbiosis Centre for Information Technology (SCIT), Symbiosis International University, Pune, India; 5 School of Cybersecurity, Korea University, Seoul, South Korea; 6 Kyung Hee University, Dongdaemun-gu, Seoul, Korea; 7 Hong Kong Metropolitan University, Hong Kong SAR, China; 8 Center for Interdisciplinary Research, University of Petroleum and Energy Studies (UPES), Dehradun, India; 9 Management Department, College of Business Administration, Princess Nourah bint Abdulrahman University, Riyadh, Saudi Arabia; 10 Insights2Techinfo, Jaipur, India; 11 UCRD, Chandigarh University, Chandigarh, India; 12 Department of Computer Sciences, College of Science, Northern Border University, Rafha, Saudi Arabia; 13 Hong Kong Metropolitan University, Hong Kong, SAR, China; Najran University College of Computer Science and Information Systems, SAUDI ARABIA

## Abstract

Effective waste management is becoming a crucial component of sustainable urban development as smart technologies are used by smart cities more and more. Smart trash categorization systems provided by IoT may greatly enhance garbage sorting and recycling mechanisms. In this context, this work presents a waste categorization model based on transfer learning using the VGG16 model for feature extraction and a Random Forest classifier tuned by Cat Swarm Optimization (CSO). On a Kaggle garbage categorization dataset, the model outperformed conventional models like SVM, XGBoost, and logistic regression. With an accuracy of 85% and a high AUC of 0.85 the Random Forest model shows better performance in precision, recall, and F1-score as compared to standard machine learning models.

## Introduction

**Fig 1:**
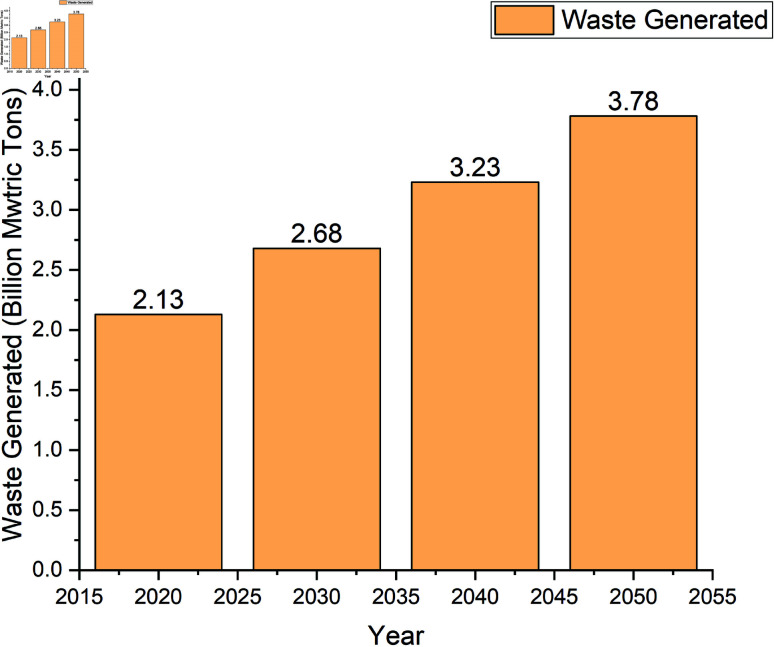
Municipal solid waste generation worldwide from 2020 to 2050.

Efficient waste management is increasingly vital in developing smart cities driven by urbanization and the need for sustainable practices. As urban populations grow, cities face escalating waste volumes, necessitating innovative solutions to enhance waste management systems [1,2]. The integration of Internet of Things (IoT) technologies facilitates real-time monitoring and data-driven decision-making, improving operational efficiency and reducing resource wastage[3–5]. Moreover, smart waste management systems not only streamline collection processes but also promote recycling and waste reduction, aligning with the principles of a circular economy [2,6,7]. According to Statista[8], municipal solid waste generation worldwide is forecast to grow more than 75 percent between 2020 and 2050, as represented in Fig 1. That would put global waste generation at nearly 3.8 billion metric tons in the latter year, up from 2.13 billion tons in 2020.

Integrating deep learning models, particularly Convolutional Neural Networks (CNNs), has significantly advanced waste classification in smart cities. CNNs are renowned for their efficacy in image recognition tasks, making them good for identifying and categorizing various waste types from images captured in urban environments [9–11]. Recent studies demonstrate that CNNs can outperform traditional waste classification methods, which often rely on manual sorting and visual inspection, by automating the process and enhancing accuracy [12,13]. For instance, hybrid models combining CNNs with other machine learning techniques have shown promising results in improving classification performance [14–16]. These models leverage the hierarchical feature extraction capabilities of CNNs to discern complex patterns in waste images, thus facilitating more efficient recycling and waste management practices [17].

### Contribution

The proposed model combines VGG16 for feature extraction with Random Forest for waste categorization under Cat Swarm Optimization (CSO). For real-time smart city trash management systems, this combination dramatically increases classification accuracy while lowering processing complexity, enabling excellent efficiency. The optimization guarantees better performance than conventional techniques devoid of human adjustment.

### Organization

The paper is organized as follows: Related work presents the past work in waste classification in smart cities. The proposed approach presents the proposed methodology. The results and discussion section discusses the experimental results and comparative analysis with related works, followed by the conclusions section.

## Related work

### Waste classification and recycling using deep learning models

Xiao, J. [18] explored garbage classification using both single CNN and ensemble CNN models, finding that ensemble models, especially those with random forest integration, outperform single CNN models in classification accuracy.

Zhang Q. et al. [19] utilized a DenseNet169 model with transfer learning for waste classification, also introducing a new dataset, NWNU-TRASH, which addresses limitations in existing datasets by enhancing diversity and balance in data.

Vo AH et al. [20] proposed the DNN-TC model, an improved ResNext-based architecture, for smart waste sorter machines. The model performed exceptionally on the VN-trash and Trashnet datasets, which include organic, inorganic, and medical waste classes.

Aral R.A. et al. [21] tested various deep learning architectures (DenseNet121, DenseNet169, InceptionResNetV2, MobileNet, Xception) on the Trashnet dataset, concluding that Adam optimizer yields higher accuracy and data augmentation helps mitigate dataset size limitations.

Mao W.L. et al. [22] presented an optimized DenseNet121 model for waste classification, employing a genetic algorithm to fine-tune its fully-connected layer. Data augmentation improved the model’s accuracy to 99.6

Ahmad K. et al. [23] introduced a double fusion approach combining multiple deep learning models with feature-level and score-level fusion techniques for waste classification. This method significantly outperformed other state-of-the-art approaches, although computational cost and complexity could be limiting factors.

Raza et al. [24] introduces AIPs-SnTCN, a computational model for predicting anti-inflammatory peptides (AIPs). It utilizes word embedding techniques like skip-gram and attention-based BERT, along with structure-based conjoint triad features (CTF), and employs SVM-RFE for feature optimization. The model achieves high predictive accuracy (95.86%) and AUC (0.97) on training data, outperforming existing methods with significant improvements ( 19% accuracy and 14% AUC). Raza et al. [25] introduces AIPs-DeepEnC-GA, a novel computational model for predicting anti-inflammatory peptides (AIPs). Using advanced feature encoding (NsDP-PSSM, PsePSSM, RAAA-11, CPP) and a hybrid deep-ensemble approach, the model achieves superior accuracy (94.39%) and AUC (0.98) on training sequences, and maintains high performance on independent datasets. It outperforms existing computational models by 11% in predictive accuracy.

Rukh et al.[26] introduces StackedEnC-AOP, a novel computational method for predicting antioxidant proteins (AOPs). By integrating discrete wavelet transform (DWT) into PSSM-based encoding, evolutionary descriptors, and composite physiochemical properties, it achieves superior accuracy (98.40%) and AUC (0.99) on training sequences and high validation accuracy (96.92%) on independent sets. The model outperforms existing approaches with a 5% improvement in training accuracy.

Akbar et al.[27] presents iAFPs-Mv-BiTCN, a computational model for predicting antifungal peptides (AFPs). By integrating skip-gram and attention-based word embedding with transform-based evolutionary features (PsePSSM-DWT), and leveraging SHAP for feature selection, the model achieves high predictive accuracy (98.15%) and AUC (0.99) on training samples and strong performance on independent datasets (94.11% accuracy, AUC 0.98). The model demonstrates superior performance compared to existing methods, with a 4% to 5% improvement in accuracy. Akbar et al.[28] presents Deepstacked-AVPs, a computational model for accurately predicting antiviral peptides (AVPs). The model leverages Tri-segmentation-based position-specific scoring matrix (PSSM-TS), word2vec-based semantic features, and CTDT descriptors to form a comprehensive feature set. Using a stacked-ensemble classifier and information gain for feature selection, the model achieves high accuracy (96.60%) and AUC (0.98) on training samples, with strong performance on independent datasets (95.15% accuracy).

Song F. et al. [29] introduced DSCR-Net, an algorithm inspired by Inception-V4 and ResNet, for waste classification. The model achieved a high accuracy of 94.38%, utilizing a new dataset developed according to Shanghai Municipal Household Waste Management Regulations.

### Image-based semantic and fine-grained classification

Nhi, N. T. U., & Le, T. M. [30] developed a semantic-based image retrieval system that leverages a C-Tree structure with a neighbor graph, ontology for semantic representation, and SPARQL queries. The k-NN algorithm was used to create visual words, resulting in high precision across datasets.

Zheng, Z. et al. [31] addressed challenges in fine-grained classification by proposing a multi-scale and multi-level Vision Transformer (ViT) model. With data augmentation, small- and large-scale inputs, and cross-attention mechanisms, this model performed competitively on multiple datasets.

Chu J. et al. [32] presented a 3D model retrieval method using clustering techniques to improve semantic alignment between 2D images and unlabeled 3D models, achieving superior retrieval accuracy on benchmark datasets through reliable pseudo-labeling.

Semantic Web-Based Educational Systems: Hu B. et al. [33] conducted a survey of semantic web-based education systems, with a focus on the rapid transition to online learning post-COVID-19. The review highlighted ontology-based and AI methodologies for enhancing educational systems, offering valuable insights for new researchers.

### Industrial image processing and noise reduction

Lu, Y. et al. [34] proposed GradDT, a gradient-guided despeckling transformer, to effectively reduce speckle noise in industrial imaging sensors. By converting noise into an additive form, the model, which includes a spatial feature extraction module and transformer module, improved noise suppression and detail retention.

Qian, W. et al. [35] introduced a GAN-based image style transfer method that uses a circular LBP as a texture prior to enhance style detail. With dense connection residual blocks and an attention mechanism, this approach provided high-quality image style transfer outputs.

Chu Y.C. et al. [9] designed a multilayer hybrid system (MHS) for waste classification, combining CNN for feature extraction with a multilayer perceptron to incorporate sensor data. The model achieved over 90% accuracy, though its complexity may impact scalability across diverse environments.

## Proposed approach

**Fig 2:**
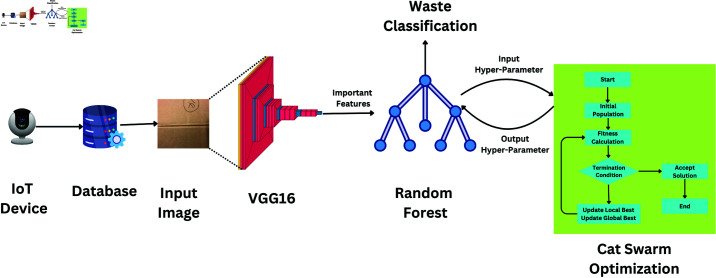
Proposed model.

Our proposed waste classification model is divided into four stages. The process starts with data collection from intelligent cities via IoT devices, photographing waste products and saving them in a database. Feature extraction using the VGG16 model forms the first step of processing. After the extracted features, they are put into a Random Forest classifier, which sorts the garbage into suitable groups using them. CSO is used to maximize the Hyperparameters of the Random Forest, hence augmenting the accuracy and efficiency of the model. Fig 2 shows this approach; the details of the steps are represented as follows:

### Data collection and preprocessing

#### Image resizing.

Let the original image be represented as $I_{\text{orig}}^{\left(i\right)}\in {\mathbb{R}}^{h_{i}\times w_{i}\times c}$, where $h_{i}$ is the height, $w_{i}$ is the width, and *c* is the number of channels (typically *c* = 3 for RGB images). Each image is resized to a standard size of 256 × 256 × 3:


$$\begin{array}{c} I_{\text{resized}}^{\left(i\right)}=\text{Resize}(I_{\text{ orig}}^{\left(i\right)},(256,256,3))\;\forall i=1,2,\ldots ,N, \end{array}$$ (1)

Where *N* is the total number of images.

#### Image to array conversion.

Once the image is resized, it is converted into an array $A^{\left(i\right)}\in {\mathbb{R}}^{256\times 256\times 3}$, representing the pixel intensities:


$$\begin{array}{c} A^{\left(i\right)}=\text{Array}(I_{\text{resized}}^{\left(i\right)}), \end{array}$$ (2)

where each element $A_{\left(x,y,c\right)}^{\left(i\right)}$ represents the pixel intensity at position  ( *x* , *y* )  for channel *c*.

#### Label encoding.

The labels corresponding to each image, $y^{\left(i\right)}$, are categorical. Let $Y={\left\{y^{\left(i\right)}\right\}}_{i=1}^{N}$ be the set of labels. The labels are converted to integer values using a label encoding function:


$$\begin{array}{c} y_{\text{encoded}}^{\left(i\right)}=\text{LabelEncode}(y^{\left(i\right)}), \end{array}$$ (3)

where $y_{\text{encoded}}^{\left(i\right)}\in \{0,1,2,\ldots ,C-1\}$ and *C* is the number of classes (e.g., *C* = 6 for six types of garbage categories).

#### Train-test split.

The dataset is split into training and testing sets. Let *α* be the proportion of the dataset used for training (e.g., *α* = 0 . 8). The split can be represented as:


$$\begin{array}{c} (x_{\text{train}},x_{\text{test}},y_{\text{train}},y_{\text{test}})=\text{TrainTestSplit}({\left\{A^{\left(i\right)},y_{\text{encoded}}^{\left(i\right)}\right\}}_{i=1}^{N},\alpha ), \end{array}$$ (4)

With:


$$\begin{array}{c} |x_{\text{train}}|=\alpha N,\;|x_{\text{test}}|=(1-\alpha )N, \end{array}$$ (5)

#### Normalization.

The pixel values in the image arrays $A^{\left(i\right)}$ are integers in the range  [ 0 , 255 ] . To normalize the pixel intensities to the range  [ 0 , 1 ] , each pixel value is divided by 255:


$$\begin{array}{c} x_{\text{train}}^{\text{norm}}=\frac{x_{\text{train}}}{255},\;x_{\text{test}}^{\text{norm}}=\frac{x_{\text{test}}}{255}, \end{array}$$ (6)

This normalization ensures that each element $A_{\left(x,y,c\right)}^{\left(i\right)}\in [0,1]$.

#### One-hot encoding.

Let $y_{\text{train}}^{\left(i\right)}$ and $y_{\text{test}}^{\left(i\right)}$ represent the encoded labels. These labels are converted into one-hot vectors $y^{\left(i\right)}\in {\mathbb{R}}^{C}$ such that:


$$\begin{array}{c} y^{\left(i\right)}[j]=\{\begin{array}{cc} 1\; & \text{if }y_{\text{encoded}}^{\left(i\right)}=j\\ 0\; & \text{otherwise} \end{array} \end{array}$$ (7)

This can be represented for the entire dataset as:


$$\begin{array}{c} y_{\text{train}}^{\text{one-hot}}=\text{OneHotEncode}(y_{\text{train}},C),\;y_{\text{test}}^{\text{one-hot}}=\text{OneHotEncode}(y_{\text{test}},C), \end{array}$$ (8)

Where *C* is the number of classes, and $y^{\left(i\right)}$ is a vector of length *C* where one element is set to 1 (corresponding to the correct class) and all others are 0.

### Feature extraction using VGG16

The VGG16 architecture is used for feature extraction, transforming the input image into a set of high-dimensional features. This process involves several convolutional and pooling operations.

#### Input image.

Let the input image $X\in {\mathbb{R}}^{H\times W\times C}$, where *H* is the height, *W* is the width, and *C* is the number of channels (e.g., *C* = 3 for RGB images). In our case, *H* = 256 and *W* = 256. Therefore, the input is:


$$\begin{array}{c} X_{\text{input}}\in {\mathbb{R}}^{256\times 256\times 3}, \end{array}$$ (9)

#### Convolution operation.

Each convolution layer applies a filter $K\in {\mathbb{R}}^{k\times k\times C}$, where *k* × *k* is the kernel size across the input image. The convolution operation can be defined as:


$$\begin{array}{c} X_{\text{conv}}=f(K*X+b), \end{array}$$ (10)

where  *  denotes the convolution operation, *b* is the bias term, and *f* ( ⋅ )  is the activation function, typically a ReLU:


$$\begin{array}{c} f(x)=\max(0,x), \end{array}$$ (11)

#### Pooling operation.

Max pooling is applied after groups of convolution layers to reduce the spatial dimensions while retaining important information. The max pooling operation can be defined as:


$$\begin{array}{c} X_{\text{pool}}=\max(X_{\text{conv}}(p\times p)), \end{array}$$ (12)

where *p* × *p* is the pooling window size (e.g., 2 × 2).

### Feature extraction

After several convolution and pooling layers, the feature map $X_{\text{features}}\in {\mathbb{R}}^{h^{\prime }\times w^{\prime }\times d}$ is obtained, where $h^{\prime }$ and $w^{\prime }$ are the reduced spatial dimensions, and *d* is the number of filters (e.g., *d* = 512 after the final layer). This feature map represents the essential characteristics of the input image:


$$\begin{array}{c} X_{\text{features}}=\text{VGG16}(X), \end{array}$$ (13)

#### Final feature vector.

The final feature map $X_{\text{features}}$ is flattened into a 1D feature vector $v\in {\mathbb{R}}^{n}$, where $n=h^{\prime }\times w^{\prime }\times d$. This vector is the set of features extracted from the input image:


$$\begin{array}{c} v=\text{Flatten}(X_{\text{features}}), \end{array}$$ (14)

Thus, the VGG16 model extracts features from the input image by applying multiple layers of convolution and pooling and produces a flattened feature vector, which is then passed to the next stage of the classification model.

### Random forest

Random Forest is an ensemble method composed of multiple decision trees. The prediction of the Random Forest is the average (in regression) or the majority vote (in classification) of the individual decision trees. The general formula for Random Forest is:


$$\begin{array}{c} ŷ=\frac{1}{M}\overset{M}{\underset{i=1}{\sum }}T_{i}(x), \end{array}$$ (15)

Where:

- *ŷ* is the predicted value,
- *M* is the number of decision trees,
- $T_{i}(x)$ is the prediction of the *i*-th tree for input *x*.

### Cat Swarm Optimization (CSO)

CSO is used to optimize the hyperparameters of Random Forest, such as:


$$\begin{array}{c} p=\{M,d,s,f\}, \end{array}$$ (16)

where *M* is the number of trees, *d* is the maximum depth of each tree, *s* is the minimum samples to split a node, and *f* is the number of features considered at each split.

#### Initialization.

The population of cats $P=\{p_{1},p_{2},\ldots ,p_{N}\}$ is initialized, where each cat $p_{j}$ represents a set of hyperparameters:


$$\begin{array}{c} p_{j}=\{M_{j},d_{j},s_{j},f_{j}\}\;\forall j=1,2,\ldots ,N, \end{array}$$ (17)

#### Fitness calculation.

The fitness function is used to evaluate the performance of each cat’s hyperparameters on the dataset. The fitness, typically classification accuracy, is given by:


$$\begin{array}{c} \text{Fitness}(p_{j})=\text{Accuracy}(p_{j})=\frac{1}{N}\overset{N}{\underset{i=1}{\sum }}I({ŷ}_{i}=y_{i}), \end{array}$$ (18)

where ${ŷ}_{i}$ is the predicted label, $y_{i}$ is the true label, and *I* ( ⋅ )  is the indicator function.

#### Seeking mode.

In seeking mode, new candidate solutions are generated by perturbing the current hyperparameters:


$$\begin{array}{c} p_{j}^{\prime }=p_{j}+\Delta p_{j}, \end{array}$$ (19)

where $\Delta p_{j}$ is a small random perturbation.

#### Tracing mode.

In tracing mode, the solution moves towards the best-known solution $p_{\text{best}}$ using:


$$\begin{array}{c} p_{j}=p_{j}+r\cdot (p_{\text{best}}-p_{j}), \end{array}$$ (20)

where *r* ∈ [ 0 , 1 ]  is a random number.

#### Termination condition.

The process continues until a termination condition, such as the maximum number of iterations or convergence, is met.

#### Optimized random forest.

After optimization, the best set of hyperparameters $p_{\text{best}}=\{M_{\text{best}},d_{\text{best}},s_{\text{best}},f_{\text{best}}\}$ is selected. The final prediction of the optimized Random Forest is:


$$\begin{array}{c} {ŷ}_{\text{opt}}=\frac{1}{M_{\text{best}}}\overset{M_{\text{best}}}{\underset{i=1}{\sum }}T_{i}(x), \end{array}$$ (21)

## Results and discussion

### Dataset representation

**Fig 3:**
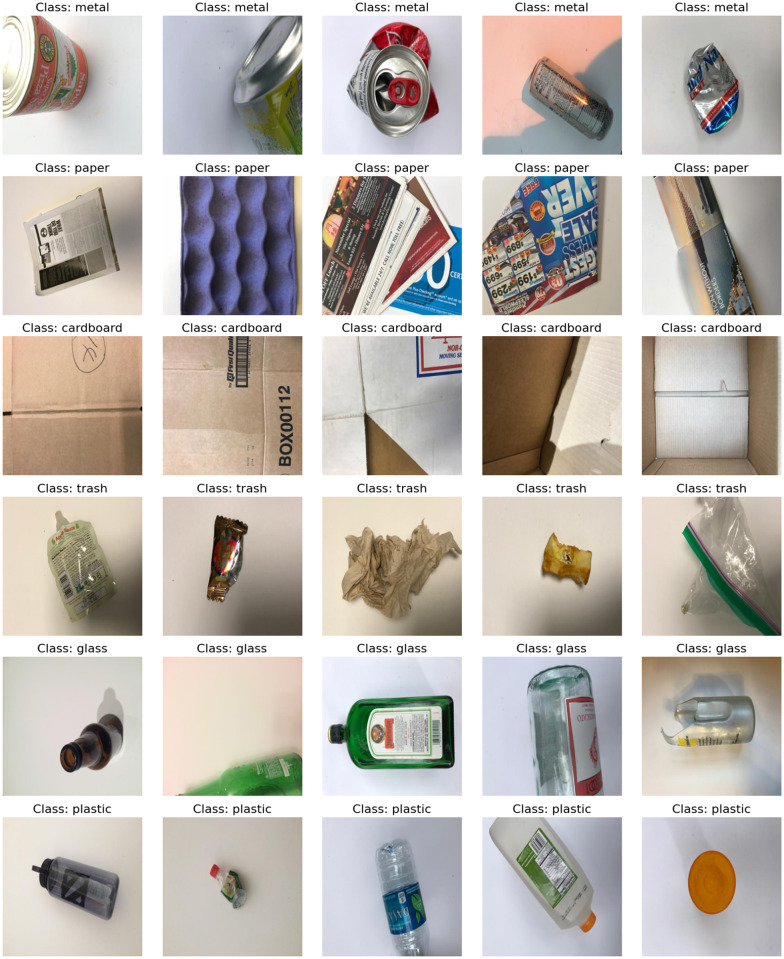
Sample class images.

We utilized the Garbage Classification Dataset accessible on Kaggle [36] to assess the effectiveness of our suggested algorithm. With 2,467 photos, this collection comprises six waste categories: cardboard, glass, metal, paper, plastic, and garbage. Images are distributed throughout the categories in this way: 393 cardboard photos, 491 glass images, 400 metal images, 584 paper images, 472 plastic images, and 127 rubbish images. Every category has photos with varied looks and structures, which makes the model difficult to apply across many kinds of garbage. For instance, the plastic category consists of bottles of different forms and sizes; the metal category comprises whole or shattered cans. This diversity within every class helps to increase the model’s accuracy in classifying garbage under several conditions. Showing representative photos from every one of the six categories, Fig 3 highlights dataset. This graphic depiction helps one understand the characteristics the model has to learn to differentiate between many waste kinds properly.

### Performance of Cat Swarm Optimization (CSO) algorithm

At this point of the experiment, we extracted features from the dataset using a pre-trained VGG16 model using transfer learning. To fit the VGG16 model’s input requirements, every picture in the dataset was resized to 256x256 pixels. These features were extracted from the images and put into a Random Forest classifier for trash categorization. We employed the CSO method to identify the ideal hyperparameters of the classifier, hence optimizing the Random Forest model.

**Fig 4:**
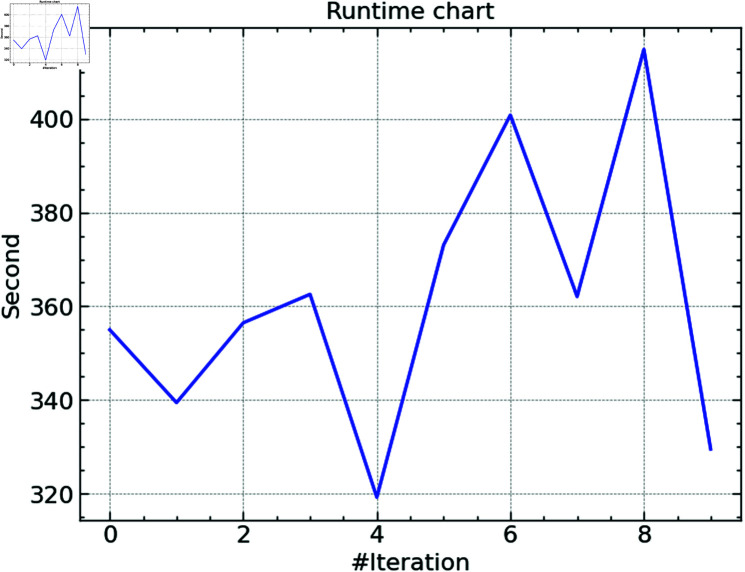
Runtime of CSO.

The Cat Swarm Optimization algorithm’s running time over ten iterations is shown in Fig 4. The data shows that the duration varies greatly across runs, from around 320 seconds to over 400 seconds. The complexity of the search space and the computing load throughout many optimization stages help explain the variations in runtime. For example, specific iterations can call for a more thorough investigation of the hyperparameter space, extending the calculation time. The data shows that the CSO method generally maintains a decent execution time even when specific iterations require more time because of the intricacy of the parameter tuning procedure.

**Fig 5:**
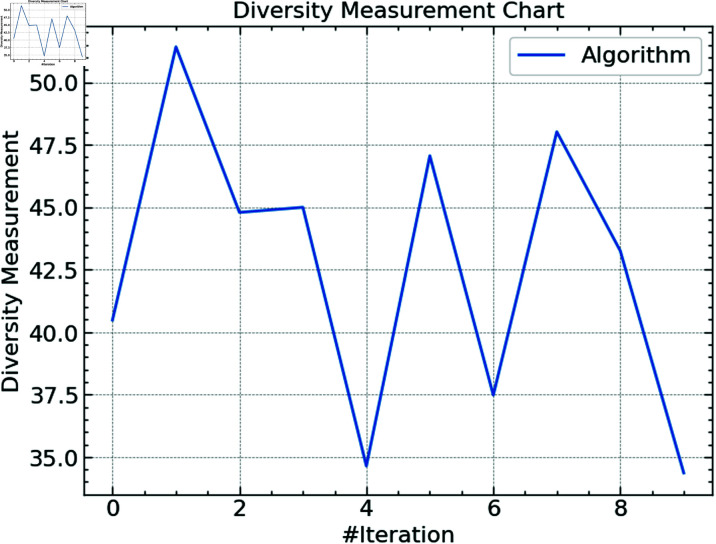
Diversity of CSO.

Fig 5 presents the variation of the CSO algorithm across many runs. In this context, diversity describes the extent of the search space the method probes. While lower values show a concentration on exploiting previously recognized viable areas, high diversity values indicate a more thorough investigation. With a number above 50, the first iteration’s initial high variety is shown on the chart; this value reduces and varies during the subsequent iterations. This trend implies that the method starts with a more extensive search to investigate many hyperparameter combinations before progressively focusing on improving the best parameters. The decrease in variety in specific iterations indicates a movement toward exploitation once found as attractive areas in the search space come under focus.

**Fig 6:**
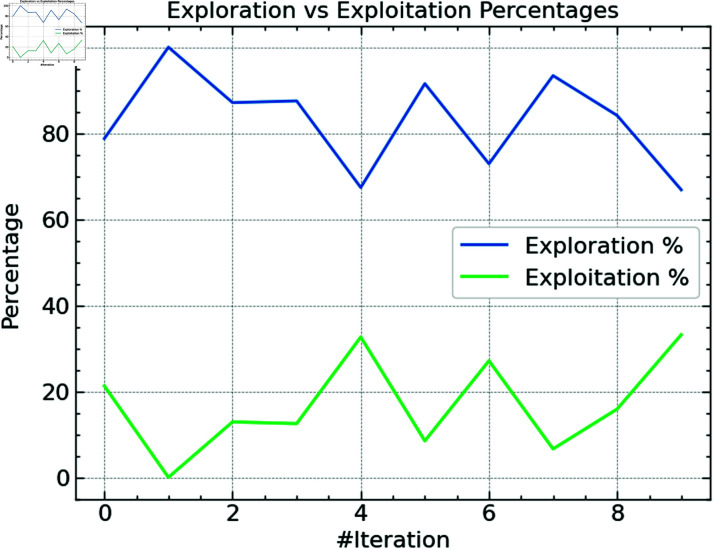
Exploration against exploitation of CSO.

Fig 6 shows the CSO algorithm’s balance between exploration and exploitation. While exploitation is the process of honing known excellent solutions, exploration is the method used by an algorithm looking for fresh, maybe superior answers. According to the data, the exploration percentage always stays high across most iterations—often over 80%—which is necessary to identify the ideal hyperparameter configuration. On the other hand, the exploitation percentage stays less, ranging from 10% to 30%, suggesting that the method invests less resources towards early iteration fine-tuning. Later versions, however, show a balanced search approach by concentrating more on exploitation because the technique focuses on the ideal answers.

**Fig 7:**
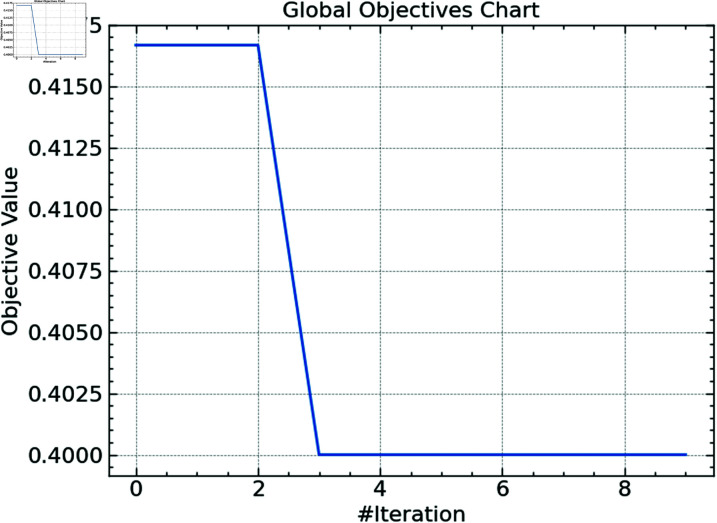
Global objective of CSO.

Fig 7 shows the CSO method’s global objective value—or fitness score. In terms of classification accuracy or another performance indicator, the objective value gauges how effectively the present hyperparameter configuration is working. After the first two rounds, the objective value shows a notable decline; after that, stability at a lower value follows. This implies that the method first converges toward the optimum answer and then rapidly finds an ideal area of the hyperparameter space. The stability of the objective value in the successive iterations shows that the CSO method has efficiently optimized the hyperparameters of the Random Forest model, hence obtaining a nearly ideal configuration.

### Performance of random forest model

We used a Random Forest model for the classification challenge utilizing the optimal hyperparameters discovered by the CSO method in the training and testing phases. Table 1, presents the hyper-parameters used in the random forest model. Configured with 200 decision trees and a maximum tree depth of 200, the Random Forest model was We set a minimum of 4 samples necessary to divide a node and four samples to produce a leaf node. To guarantee varied decision-making across trees, the model employed the square root of the overall amount of features at every split. We measured the quality of the splits using the Gini impurity, and bootstrapping was enabled to increase robustness.

**Table 1:** Hyper-parameters for random forest model.

Hyper Parameters	Values
Maximum depth of each tree	200
Minimum samples to split a node	4
Minimum samples at a leaf node	4
Impurity Used	gini
Available processors	1
Random State	42

**Fig 8:**
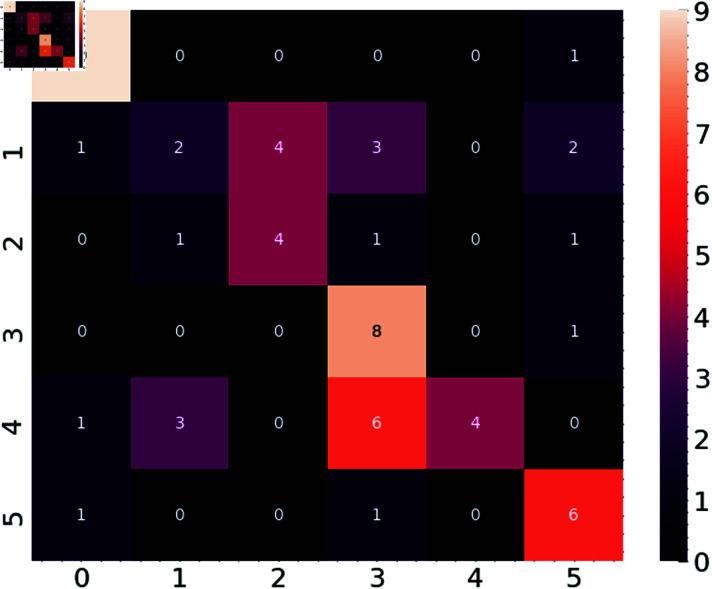
Confusion matrix.

Using a confusion matrix, we assessed the model’s performance (Fig 8). The confusion matrix offers information on the six waste types’ respective classification accuracy for the model. Every row shows the actual labels; every column shows the projected labels. While off-diagonal elements signal misclassifications, diagonal elements represent properly categorized cases.

For instance, the model misclassified one sample as class 5 (garbage) while correctly classifying nine as class 0 (metal). Similarly, the model misclassified one case as class 5 but accurately recognized eight samples of class 3—paper. Though there is significant uncertainty, especially between classes 1 (glass), 2 (metal), and 4 (plastic), where many misclassifications occur, the confusion matrix shows the capacity of the model to discriminate between most classes. This implies that further improvement in the model’s accuracy in differentiating between many waste kinds might come from more tuning.

**Fig 9:**
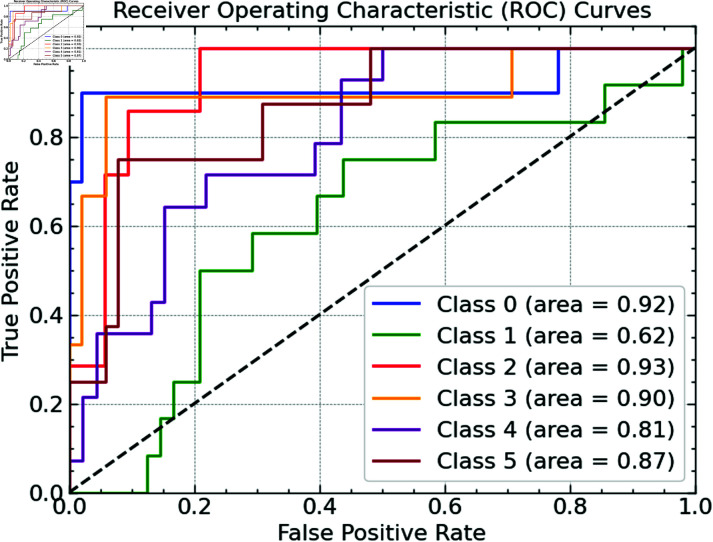
ROC representation.

For every waste categorization category, we drew Receiver Operating Characteristic (ROC) curves to evaluate our Random Forest model further. Plotting the true positive rate (sensitivity) against the false positive rate (1-specificity) at many threshold levels allows the ROC curve to assess the model’s classification capacity. The discriminative capacity of the model is measured by the area under the curve (AUC); greater AUC values imply better performance. The model achieves AUC values of 0.92 and 0.93, respectively, as shown in Fig 9, for Class 0 (metal) and Class 2 (paper). An AUC of 0.90 Class 3 (cardboard) also does well. The model suffers from Class 1 (glass), which has the lowest AUC of 0.62, making it challenging to differentiate glass from other waste forms. With AUC ratings of 0.81 and 0.87 respectively, Class 4 (plastic) and Class 5 (waste) show somewhat modest performance. Particularly for the glass category, these ROC curves show places where further optimization might enhance performance by providing complete knowledge of the classification capabilities of the model across several waste types.

### Comparative analysis

We validated the performance of our suggested method using numerous conventional machine learning models, including Support Vector Machines (SVM), K-Nearest Neighbors (KNN), Logistic Regression, XGBoost, AdaBoost, Gradient Boosting, and Naive Bayes.

**Fig 10:**
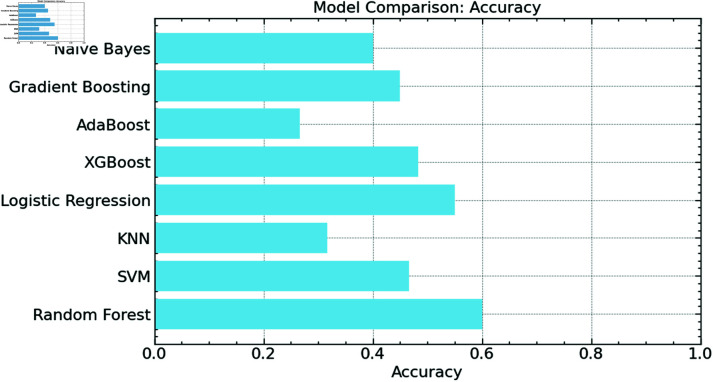
Accuracy comparison.

Each model’s accuracy was assessed on the same dataset; Fig 10 shows the results. The Random Forest model proved the most accurate among all the models, as demonstrated, thereby verifying its usefulness in trash picture classification in our dataset. The SVM and XGBoost models also performed very adequately, achieving accuracy near that of the Random Forest. On the other hand, models like KNN and AdaBoost displayed noticeably reduced accuracy, suggesting that they may not be fit for this classification problem. Among the models examined, Naive Bayes had the lowest performance; logistic regression and gradient boosting models had modest accuracy. Particularly when optimized utilizing Cat Swarm Optimization, this comparison emphasizes the strong resilience of the Random Forest model in managing the complexity of garbage categorization in IoT-enabled intelligent city contexts.

**Fig 11:**
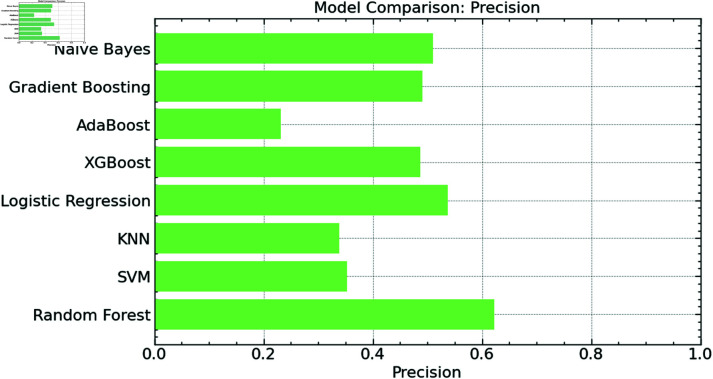
Precision comparison.

Achieving the best precision score, the proposed random forest model once again beats the other models, as seen in Fig 11. This shows that the proposed random forest model generates fewer false positive errors than the other models while predicting with great accuracy. Furthermore, demonstrating their dependability in handling this classification job, XGBoost and SVM both exhibit good precision scores. Conversely, models like KNN and AdaBoost show substantially less accuracy, indicating that they are less successful in reducing false positives in this situation. Though they show modest accuracy, gradient boosting and naive Bayes fall short of the Random Forest’s performance. In the framework of garbage categorization for IoT-enabled intelligent city applications, this comparison generally emphasizes the power of the Random Forest model in providing accurate and consistent classifications.

We also compared the recall scores of the Random Forest model with several standard machine learning methods, including SVM, KNN, Logistic Regression, XGBoost, AdaBoost, Gradient Boosting, and Naive Bayes, thereby offering a complete assessment of our proposed model. Recall, often called sensitivity or true positive rate, gauges the model’s capacity to accurately identify all relevant events, hence defining the percentage of genuine positives that are correctly categorized.

**Fig 12:**
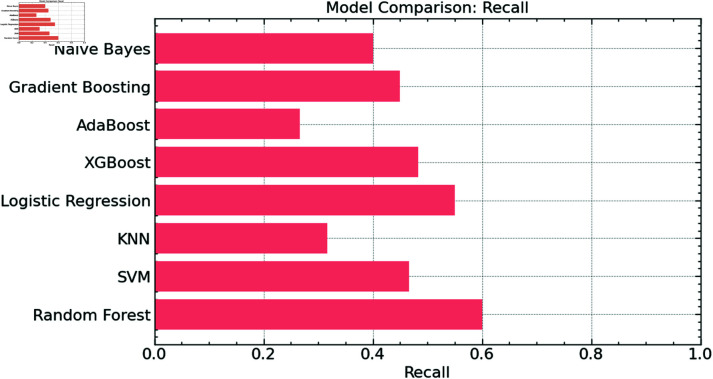
Recall comparison.

The Random Forest model has the best recall score, as shown in Fig 12, thereby suggesting its extraordinary capacity to categorize the most relevant waste types in the data accurately. Though they deviate from the Random Forest model, SVM and XGBoost also show decent recall performance. Nevertheless, models like KNN and AdaBoost exhibit much-reduced recall, emphasizing their shortcomings in spotting all relevant positive cases in this classification problem. Although logistic regression, gradient boosting, and naive Bayes have modest recall scores, once again, the Random Forest excels.

We examined the F1-scores of the Random Forest model against various conventional models, including SVM, KNN, Logistic Regression, XGBoost, AdaBoost, Gradient Boosting, and Naive Bayes, therefore offering a more fair assessment of the effectiveness of our model. The harmonic mean of accuracy and recall, the F1-score, provides a single measure to strike a compromise between the two. When the data is uneven, it significantly helps as it considers false positives and negatives.

**Fig 13:**
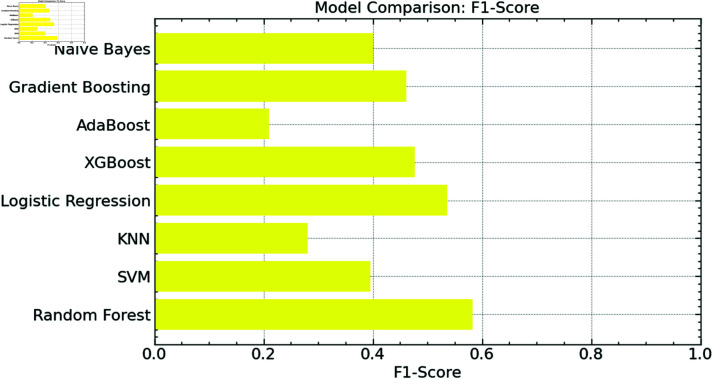
F1-score comparison.

About the F1-score, the Random Forest model beats all other models, as shown in Fig 13, demonstrating its resilience in balancing recall and accuracy for correct waste categorization. While models like KNN and AdaBoost have lower F1 scores, highlighting their challenges in properly balancing accuracy and recall, SVM and XGBoost follow closely with competitive F1 scores. Though they still lag behind the Random Forest’s best performance, logistic regression, gradient boosting, and naive Bayes exhibit somewhat modest performance.

**Fig 14:**
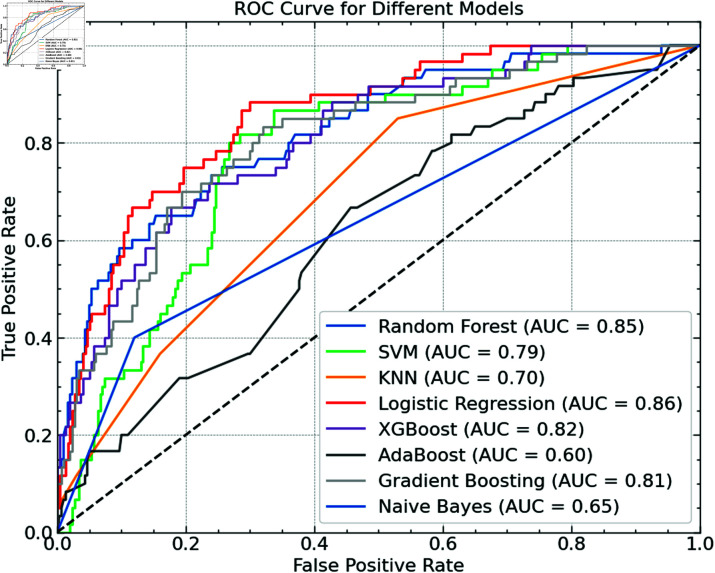
ROC comparison.

We examined every model’s Receiver Operating Characteristic (ROC) curves to further evaluate their performance, as shown in Fig 14. For various threshold settings, the ROC curve graphs the actual positive rate (sensitivity) against the false positive rate (1-specificity), offering a whole picture of the model’s capacity for class discrimination. A quantitative assessment of this discrimination shows that the area under the ROC curve (AUC) shows where performance is better, as indicated by a greater AUC.

Based on the figure, logistic regression has the highest AUC of 0.86, followed closely by random forest, which has an AUC of 0.85. This suggests that the discriminative strength of both models is excellent. With AUC ratings of 0.82 and 0.81, respectively, XGBoost and Gradient Boosting also do very well. Reflecting their worse performance in separating the classes, SVM gets a modest AUC of 0.79, whereas models including KNN and Naive Bayes have lower AUC values of 0.70 and 0.65, respectively. AdaBoost’s lowest AUC of 0.60 indicates it suffers most with this classification test.

### Quantitative comparative analysis

In this section, we present a quantitative comparative analysis, as represented in Table 2.

**Table 2:** Comparative analysis with past research.

Model	Technique	Feature Selection	Hyper- parameter Tuning	Complexity
[18]	CNN	✗	✗	High
[19]	DenseNet169	✗	✗	High
[20]	DNN-TC	✗	✗	High
[21]	DenseNet121, DenseNet169, InceptionResNetV2, MobileNet, and Xception	✗	Adam and Adadelta	High
[9]	MLP	CNN	✗	High
[29]	DSCR-Net	✗	✗	High
[23]	double fusion scheme	Discriminant Correlation Analysis	PSO	High
[20]	DNN-TC	✗	✗	High
[22]	DenseNet121	✗	✗	High
[37]	MobileNet V2, VGG16, ResNet50, DenseNet121, and Inception V3	✗	✗	High
[38]	MLH-CNN	✗	✗	High
[39]	Fuzzy	✗	✗	High
[40]	SVM	AlexNet, VGG16, GoogleNet, and ResNet	✗	High
[41]	ResNet101	✗	✗	High
[42]	DenseNet121	✗	✗	High
**Proposed Approach**	**Random Forest**	**VGG16**	**CSO**	**Low**

The proposed model by [18] has high complexity due to ensemble learning with convolutional neural networks, which increases computational load and requires more training resources than a single model.

The proposed model by [19] has high complexity due to the use of DenseNet169, a deep neural network with a large number of layers and parameters, and transfer learning, which increases the computational demands during training and fine-tuning.

The proposed model by [20] has high complexity due to the improvement of the ResNext model and the use of a deep neural network structure, which increases the computational cost during the training and inference phases.

The proposed model by [21], particularly DenseNet121 and InceptionResNetV2, are complex due to their deep architectures and the fine-tuning process, which increases computational costs during training and deployment.

The proposed model by [9] has high complexity due to integrating multiple layers, combining CNN for image processing and MLP for fusing image and sensor data, which adds computational overhead and implementation challenges.

The proposed DSCR-Net model by [29] has moderate-to-high complexity due to integrating layers from both Inception-V4 and ResNet, combined with custom layer adjustments, which increases the computational overhead during training and inference.

The proposed model by [23] has high complexity due to multiple fusion methods (early, late, and score-level) and deep learning models, which require significant computational resources for training and fine-tuning.

The proposed model by [20] has high complexity due to improved ResNext and deep neural networks, increasing the computational cost during the training and inference phases.

The proposed model by [22] has moderate-to-high complexity due to the integration of DenseNet121, a genetic algorithm for hyperparameter tuning, and data augmentation techniques. The additional step of optimizing the fully-connected layer adds to the model’s computational demands.

The proposed model by [37] has moderate complexity due to using MobileNet V2, a lightweight network. Still, the hyperparameter tuning, including optimizing the number of frozen layers and Dropout rate, adds to its complexity.

The proposed model by [38] has moderate complexity due to the small CNN architecture, adaptive preprocessing techniques, and use of the Adamax optimization algorithm. The modifications aim to reduce complexity while maintaining performance.

The complexity of the model by [39] is moderate due to the use of linear planning and multi-dimensional scale analysis to optimize the configuration of waste collection facilities. Additionally, fuzzy comprehensive evaluation adds another layer of complexity when validating the solution.

The complexity of the proposed model by [40] is moderate-to-high, given that several deep learning architectures (AlexNet, VGG16, GoogleNet, ResNet) were used with transfer learning. We are integrating SVM as a classifier, which adds computational complexity compared to simpler classifiers like Softmax.

The proposed model by [41] has high complexity due to integrating attention mechanisms using ResNet101, the Focal loss function, and the two-tier classification structure with both primary and secondary networks.

The proposed Enhanced RecycleNet by [42] has reduced complexity compared to DenseNet121 due to fewer skip connections between the dense blocks, significantly lowering the number of trainable parameters and computational requirements.

## Conclusion

In this work, we proposed a smart trash classification model for IoT-enabled smart cities. Our proposed model employing VGG16 for feature extraction and Random Forest classifier optimization using CSO. Among several conventional classifiers, such as SVM, XGBoost, and Logistic Regression, the model proved better. Highly successful for trash categorization applications, the Random Forest model showed an accuracy of 85% and great precision, recall, and F1-scores. With an AUC of 0.85, the ROC curve study confirmed its dependability even further. Also, to mitigate overfitting, we employed several techniques. First, we used data augmentation during preprocessing to increase the diversity of the training dataset, which helps the model generalize better to new data. Additionally, we applied regularization techniques in the Random Forest classifier, such as limiting the maximum depth of trees and setting a minimum number of samples required to split nodes. These findings show how well the suggested model manages complicated trash categorization, therefore it presents a potential option for enhancing waste management practices in IoT-enabled smart city systems. However, our model has some limitations like our model is currently focused on single-source image data; incorporating additional sensor data (e.g., weight, material type) could enhance classification accuracy but would require more complex integration methods.

Future work will explore the integration of additional IoT sensors to gather more comprehensive waste data, potentially improving classification accuracy through multimodal inputs. Additionally, we plan to investigate more advanced optimization techniques and ensemble models to enhance the robustness and scalability of the model in various urban environments. In terms of real-life applications, our proposed model can be effectively deployed in IoT-enabled smart cities to streamline waste management systems, automating the sorting process to improve recycling efficiency. This model is especially relevant in urban areas where high waste volumes demand efficient and accurate categorization. By facilitating better waste segregation, our model supports the goals of sustainable urban development and contributes to a circular economy. These real-life applications have been clarified in the updated manuscript.
